# The Law of the Iterated Logarithm for Linear Processes Generated by a Sequence of Stationary Independent Random Variables under the Sub-Linear Expectation

**DOI:** 10.3390/e23101313

**Published:** 2021-10-07

**Authors:** Wei Liu, Yong Zhang

**Affiliations:** School of Mathematics, Jilin University, 2699 Qianjin Street, Changchun 130012, China; wliu18@mails.jlu.edu.cn

**Keywords:** the law of the iterated logarithm, linear process, stationary sequences, capacity, sub-linear expectation, 60F15, 60F05

## Abstract

In this paper, we obtain the law of iterated logarithm for linear processes in sub-linear expectation space. It is established for strictly stationary independent random variable sequences with finite second-order moments in the sense of non-additive capacity.

## 1. Introduction

As fundamental limit theorems of probability theory, the classical law of the iterated logarithm (LIL for short) plays an important role in the development of probability theory and its applications. The original statement of LIL obtained by Khinchine [1] is for a class of Bernoulli random variables. After that, a lot of literature has performed in-depth and detailed research on LIL, we can refer to Hartman and Wintner [2], Acosta [3], Shao and Su [4], and so on. Motivated by modeling uncertainty in practice, Peng [5] introduced the reasonable framework of the sub-linear expectation of random variables in a general function space. As an alternative to the traditional probability/expectation, capacity/sub-linear expectation has been studied in many fields, such as statistics, mathematical economics, measures of risk, and super-hedging in finance. In recent years, after studying the limit theorem of sub-linear expectation (e.g., see Feng [6], Deng and Wang [7], Tan and Zong [8], and Zhang [9,10], etc.), more and more research results of LIL under this framework have been obtained, the Hartman–Winter LIL were established by Chen and Hu [11] for bounded random variables, the functional central limit and Chung’s LIL were recently obtained by Zhang [12], and the LIL for independent and negatively dependent identically distributed random variables were proven by Zhang [13].

As is well known, the linear processes are especially important in time series analysis and they arise from a wide variety of contexts (cf. Hannan [14]). Applications to economics, engineering, and physical sciences are extremely broad and a vast amount of literature is devoted to the study of linear processes under a variety of circumstances. The limit theory of linear processes has been studied in detail in many papers. Philips and Solo [15] prove the strong law of numbers and the law of iterated logarithm for linear processes, Zhang [16] gives the limit law of the iterated logarithm for linear processes. Recently, Liu and Zhang [17] obtained the central limit theorem and invariance principle for linear processes generated by independent and identically distributed (IID for short) random variables under sub-linear expectation.

A natural question is: can LIL of linear processes be realized under Peng’s framework? The main purpose of this paper is to establish the law of iterated logarithm for linear processes generated by IID random variables in sub-linear expectation space. In the classical case, the LIL of partial sum is established by decomposing the linear process. We will find that this way is also valid for proving LIL for linear process in the sub-linear expectation space, though there are some differences. Intuitively, sub-linear expectation and related non-additive probabilities (Capacities) generated by them plays a decisive role in our proof. In the sequel, *c* denotes a positive constant, which may take different values whenever it appears in different expressions.

To state the result, we shall first recall the framework of sub-linear expectations. We use the framework and notation of Peng [5,18,19]. Let (Ω,F) be a given measurable space. Let H be a linear space of real functions defined on (Ω,F), such that if $X_{1},X_{2},...,X_{n}\in H$ then $\varphi (X_{1},X_{2},...,X_{n})\in H$ for each $\varphi \in C_{l,Lip}\left(R^{n}\right)$ where $\varphi \in C_{l,Lip}\left(R^{n}\right)$ denotes the linear space of local Lipschitz continuous functions φ satisfying

$$\left|\varphi \left(x\right)-\varphi \left(y\right)\right|\leq {c(1+|x|}^{m}+{\left|y\right|}^{m})|x-y|,\;\;\forall x,y\in R^{n},$$

for some c>0,m∈N depending on φ. H contains all $I_{A}$ where A∈F. We also denote $\varphi \in C_{b,Lip}\left(R^{n}\right)$ as the linear space of bounded Lipschitz continuous functions φ satisfying

$$\begin{array}{c} \left|\varphi \left(x\right)-\varphi \left(y\right)\right|\leq c|x-y|,\;\;\forall x,y\in R^{n}, \end{array}$$

for some c>0.

**Definition** **1.** *A function $\hat{E}:H\to \left[-\infty ,+\infty \right]$ is said to be a sub-linear expectation if it satisfies for ∀X,Y∈H,*

*1.*  *Monotonicity: X≥Y implies $\hat{E}\left[X\right]\geq \hat{E}\left[Y\right]$;*
*2.*  *Constant preserving: $\hat{E}\left[c\right]=c,\;\forall c\in R$;*
*3.*  *Sub-additivity: $\hat{E}\left[X+Y\right]\leq \hat{E}\left[X\right]+\hat{E}\left[Y\right]$;*
*4.*  *Positive homogeneity: $\hat{E}\left[\lambda X\right]=\lambda \hat{E}\left[X\right],\;\forall \lambda \geq 0$.*

The triple $\left(\Omega ,H,\hat{E}\right)$ is called a sub-linear expectation space. Give a sub-linear expectation $\hat{E}$, let us denote the conjugate expectation $\hat{E}$ of $\hat{E}$ by $\hat{E}\left[X\right]:=-\hat{E}\left[-X\right],\;\forall X\in H$.

**Remark** **1.** *(i) The sub-linear expectation $\hat{E}\left[\cdot \right]$ satisfies translation invariance: $\hat{E}\left[X+c\right]=\hat{E}\left[X\right]+c,\;\forall c\in R$. (ii) From the definition, it is easy to show that $\hat{E}\left[X\right]\leq \hat{E}\left[X\right]$ and $\hat{E}\left[X-Y\right]\geq \hat{E}\left[X\right]-\hat{E}\left[Y\right],\;\forall X,Y\in H$ with $\hat{E}\left[Y\right]$ being finite.*

**Definition** **2.** *(i) (Identical distribution) Let $X_{1}$ and $X_{2}$ be two n-dimensional random vectors defined, respectively, in sub-linear expectation spaces $\left(\Omega_{1},H_{1},{\hat{E}}_{1}\right)$ and $\left(\Omega_{2},H_{2},{\hat{E}}_{2}\right)$. They are called identically distributed, denoted by $X_{1}\overset{d}{=}X_{2}$, if*

$${\hat{E}}_{1}\left[\varphi \left(X_{1}\right)\right]={\hat{E}}_{2}\left[\varphi \left(X_{2}\right)\right],\;\forall \varphi \in C_{l,Lip}\left(R^{n}\right),$$

*whenever the sub-expectations are finite. A sequence of random variables $\left\{X_{n},n\geq 1\right\}$ is said to be identically distributed if $X_{i}\overset{d}{=}X_{1}$ for each i≥1.*

*(ii) (Independence) In a sub-linear expectation space $\left(\Omega ,H,\hat{E}\right)$, a random vector $Y=\left(Y_{1},...,Y_{n}\right)\left(Y_{i}\in H\right)$ is said to be independent to another random vector $X=\left(X_{1},...,X_{m}\right)\left(X_{i}\in H\right)$ under $\hat{E}$ if for each test function $\varphi \in C_{l,Lip}\left(R^{m}\times R^{n}\right)$ we have*

$$\hat{E}\left[\varphi \left(X,Y\right)\right]=\hat{E}\left[\hat{E}\left[\varphi \left(x,Y\right)\right]{|}_{x=X}\right],$$

*whenever $\bar{\varphi }\left(x\right):=\hat{E}\left[|\varphi \left(x,Y\right)|\right]<\infty$ for all x and $\hat{E}\left[\right|\bar{\varphi }\left(x\right)\left|\right]<\infty$.*

*(iii) (IID random variables) A sequence of random variables $\left\{X_{n},n\geq 1\right\}$ is said to be independent and identically distributed (IID), if $X_{i}\overset{d}{=}X_{1}$ and $X_{i+1}$ is independent to $\left(X_{1},...,X_{i}\right)$ for each i≥1.*

It is obvious that, if $\left\{X_{n},n\geq 1\right\}$ is a sequence of independent random variables and $f_{1}\left(x\right),f_{2}\left(x\right),...\in C_{l,Lip}\left(R\right)$, then $\left\{f_{n}\left(X_{n}\right),n\geq 1\right\}$ is also a sequence of independent random variables.

Next, we introduce the capacities corresponding to the sub-linear expectations.

**Definition** **3** ([11])**.** *A set function V: F→[0,1] is called a capacity, if*

*1.*  *V(∅)=0,V(Ω)=1;*
*2.*  *V(A)≤V(B),∀A⊂B,A,B⊂F.*

*It is called to be sub-additive if V(A∪B)≤V(A)+V(B) for all A,B∈F with A∪B∈F.*

A sub-linear expectation $\hat{E}$ could generate a pair (V,V) of capacity denoted by

$$V\left(A\right):=\inf\left\{\hat{E}\left[\xi \right]:\;I_{A}\leq \xi ,\;\xi \in H\right\},\;\;\;V\left(A\right)=1-V\left(A^{c}\right),\;\forall A\in F,$$

where $A^{c}$ is the complement set of *A*. Then

$$V\left(A\right):=\hat{E}\left[I_{A}\right],\;\;\;\;V\left(A\right):=\hat{E}\left[I_{A}\right],\;\;\;\;\mathrm{if}\;\;I_{A}\in H,$$

(1) $$\begin{array}{c} \hat{E}\left[f\right]\leq V\left(A\right)\leq \hat{E}\left[g\right],\;\;\;\;\hat{E}\left[f\right]\leq V\left(A\right)\leq \hat{E}\left[g\right],\;\;\;\;\mathrm{if}\;f\leq I_{A}\leq g,\;\;\;\;f,g\in H. \end{array}$$

In addition, a pair $\left(C_{V},\;C_{V}\right)$ of the Choquet integrals/expecations denoted by

$$\begin{array}{c} C_{V}\left[X\right]=\int_{0}^{\infty }V\left(X\geq t\right)dt+\int_{-\infty }^{0}\left[V\left(X\geq t\right)-1\right]dt, \end{array}$$

with *V* being replaced by V and V, respectively.

If $\lim_{c\to \infty }\hat{E}{\left[(|X|-c\right)}^{+}]=0$ or $\hat{E}$ is countably sub-additive, then $\hat{E}\left[|X|\right]\leq C_{V}\left(|X|\right)$ (See Lemma 4.5 (iii) of Zhang [13]).

**Definition** **4** ([20])**.** *(a) A sub-linear expectation $\hat{E}:H\to \left[-\infty ,+\infty \right]$ is called to be countably sub-additive if it satisfies $\hat{E}\left[X\right]\leq \sum_{n=1}^{\infty }\hat{E}\left[X_{n}\right],$ whenever $X\leq \Sigma_{n=1}^{\infty }X_{n},\;X,\;X_{n}\in H$ and $X\geq 0,\;X_{n}\geq 0,\;n=1,2,...$;*

*(b) A function V: F→[0,1] is called to be countably sub-additive if $V\left({⋃}_{n=1}^{\infty }A_{n}\right)\leq \sum_{n=1}^{\infty }V\left(A_{n}\right),\;\forall A_{n}\in F;$*

*(c) A capacity V: F→[0,1] is called a continuous capacity if it satisfies:*

*c1. Continuity from below: $V\left(A_{n}\right)↑V\left(A\right)$, if $A_{n}↑A$, where $A_{n},\;A\in F$;*

*c2. Continuity from above: $V\left(A_{n}\right)↓V\left(A\right)$, if $A_{n}↓A$, where $A_{n},\;A\in F$.*

It is obvious that the continuity from above and sub-additivity imply the continuity from below, and the continuity from the below and sub-additivity imply the countable sub- additivity. Therefore, we call a sub-additive capacity to be continuous if it is continuous from above. Set $H=\{A:\;I_{A}\in H\}$, then V is a countably sub-additive capacity in H if $\hat{E}$ is countably sub-additive in H, and (V,V) is a pair of continuous capacities in H if $\hat{E}$ is continuous in H.

## 2. Main Results

In this section, we shall study the LIL of linear processes under association assumption in the sub-linear expectation space. For any I∈(k,+∞), $\left\{X_{j},\;j\in I\right\}$ is a sequence of independent random variables satisfying Definition 2; For a finite index set I∈(−∞,k), $\left\{X_{j},\;j\in I\right\}$ is also a sequence of independent random variables satisfying Definition 2.

First, we give the definition of strictly stationary sequence under the sub-linear expectation.

**Definition** **5.** *$\left\{\varepsilon_{n},\;n\in N\right\}$ is said to be a sequence of strictly stationary random variables on the $\left(\Omega ,H,\hat{E}\right)$, if for any a function $\phi_{n}\in C_{l,Lip}\left(R^{n}\right):R^{n}\to R$, then*

$$\hat{E}\left[\phi_{n}\left(\varepsilon_{1},\varepsilon_{2},...,\varepsilon_{n}\right)\right]=\hat{E}\left[\phi_{n}\left(\varepsilon_{1+k},\varepsilon_{2+k},...,\varepsilon_{n+k}\right)\right],\;\;\;\;\forall n\geq 1,\;k\in N.$$

Next we give the main results: the law of the iterated logarithm for linear processes in the sub-linear expectation space.

**Theorem** **1.** *Suppose that $\left\{\varepsilon_{j},\;j\in Z\right\}$ is a sequence of strictly stationary independent random variables on the $\left(\Omega ,H,\hat{E}\right)$ with $\hat{E}\left[\varepsilon_{1}\right]=\hat{E}\left[-\varepsilon_{1}\right]=0$ and ${\bar{\sigma }}^{2}=\hat{E}\left[\varepsilon_{1}^{2}\right],\;{\underline{\sigma }}^{2}=\hat{E}\left[\varepsilon_{1}^{2}\right].$ Further, assume that $\hat{E}$ is countably sub-additive and*

$\left(A_{1}\right)$

$\hat{E}\left[\varepsilon_{1}^{2}\left(\log\right|\varepsilon_{1}{\left|\right)}^{1+\delta }\right]<\infty ,\;\;for\;some\;\delta >0;$

$\left(A_{2}\right)$

$\lim_{c\to \infty }\hat{E}\left[{\left(\varepsilon_{1}^{2}-c\right)}^{+}\right]=0;$

$\left(A_{3}\right)$

$C_{V}\left[\frac{\varepsilon_{1}^{2}}{\log\log|\varepsilon_{1}|}\right]<\infty .$

*Define the linear process by $X_{t}=\sum_{j=-\infty }^{\infty }\alpha_{j}\varepsilon_{t-j},\;t\geq 1$ and the partial sum $T_{n}=\sum_{t=1}^{n}X_{t}$, where $\left\{\alpha_{j},\;j\in Z\right\}$ is a sequence of real numbers satisfying $A=|\sum_{j=-\infty }^{\infty }\alpha_{j}|\neq 0,$ $\sum_{j=-\infty }^{\infty }\left|\alpha_{j}\right|<\infty .$ Then we have*

(2) $$\begin{array}{c} V\left(\underset{n\to \infty }{lim sup}\frac{|T_{n}|}{a_{n}}\leq A\bar{\sigma }\right)=1, \end{array}$$

*where $a_{n}=\sqrt{2n\log\log n}$, logn=ln(n∨e), $\log\log n=\ln\ln(n\vee e^{e}),\;n\geq 1$.*

**Remark** **2.** *If $\alpha_{0}=1,\;\alpha_{j}=0,\;j\neq 0$, Theorem 1 can be regarded as Lemma 3.*

**Remark** **3.** *In particular, according to Proposition 4.1 in Zhang [10], for the random variable sequence of IID, if V is continuous, then $\hat{E}$ is linear. Then, the LIL of this paper is the known result of classical probability space.*

## 3. Proofs

In order to prove the main results, we need the following Lemmas. The first one was the convergence part of the Borel–Cantelli Lemma.

**Lemma** **1** ([20])**.** *Let $\left\{A_{n},n\geq 1\right\}$ be a sequence of events in F. Suppose that V is a countably sub-additive capacity. If $\sum_{n=1}^{\infty }V\left(A_{n}\right)<\infty$ then*

$$V\left(A_{n}\;i.o.\right)=0,\;\;\;\;where\;\left\{A_{n}\;i.o.\right\}=\overset{\infty }{\underset{n=1}{⋂}}\overset{\infty }{\underset{i=n}{⋃}}A_{i}.$$

The second Lemma on the exponential inequality is Lemma 2.1 of Zhang [9].

**Lemma** **2** ([9])**.** *Let $\left\{Z_{n,k}:k=1,...,k_{n}\right\}$ be an array of independent random variables, such that $\hat{E}\left[Z_{n,k}\right]\leq 0$ and $\hat{E}\left[Z_{n,k}^{2}\right]<\infty ,\;k=1,...,k_{n}$. Then for all x,y>0*

(3) $$\begin{array}{cc} & \;\;\;\;V\left(\max_{m\leq k_{n}}\sum_{k=1}^{m}Z_{n,k}\geq x\right)\\ \; & \leq V\left(\max_{k\leq k_{n}}Z_{n,k}\geq y\right)+\exp\left\{\frac{x}{y}-\frac{x}{y}\left(\frac{B_{n}}{xy}+1\right)\ln\left(1+\frac{xy}{B_{n}}\right)\right\}, \end{array}$$

*where $B_{n}=\sum_{k=1}^{k_{n}}\hat{E}\left[Z_{n,k}^{2}\right]$.*

The following Lemma is a law of iterated logarithm under sub-linear expectation.

**Lemma** **3.** *Let $\left\{\varepsilon_{n},\;n\geq 1\right\}$ be a sequence of IID random variables in $\left(\Omega ,\;H,\;\hat{E}\right)$ with $\hat{\hat{E}}\left[\varepsilon_{1}\right]=\hat{E}\left[-\varepsilon_{1}\right]=0$. Write $\hat{E}\left[\varepsilon_{1}^{2}\right]={\bar{\sigma }}^{2},\;E\left[\varepsilon_{1}^{2}\right]={\underline{\sigma }}^{2}$, $S_{n}=\sum_{k=1}^{n}\varepsilon_{k}$. Suppose that the conditions $\left(A_{2}\right)$ and $\left(A_{3}\right)$ in Theorem 1 hold. If V is countably sub-additive, then we have*

(4) $$\begin{array}{c} V\left(\underset{n\to \infty }{lim sup}\frac{|S_{n}|}{a_{n}}\leq \bar{\sigma }\right)=1. \end{array}$$

**Proof.**  Obviously, (4) can be directly derived from Theorem 3.11 and (4.29) in Zhang [13]. □

**Lemma** **4.** *Let $\left\{\varepsilon_{n},\;n\in Z\right\}$ be a sequence of IID random variables on the $\left(\Omega ,H,\hat{E}\right)$ with $\hat{E}\left[\varepsilon_{1}\right]=\hat{E}\left[-\varepsilon_{1}\right]=0$ and ${\bar{\sigma }}^{2}=\hat{E}\left[\varepsilon_{1}^{2}\right],\;{\underline{\sigma }}^{2}=\hat{E}\left[\varepsilon_{1}^{2}\right]$. Further assume that $\hat{E}$ is countably sub-additive and the condition $\left(A_{1}\right)$ in Theorem 1 hold. Then we have*

(5) $$\begin{array}{c} \hat{E}\left[\underset{n}{\sup}{\left(2n\log\log n\right)}^{-1/2}\left|\sum_{k=1}^{n}\varepsilon_{k}\right|\right]<\infty . \end{array}$$

**Proof.**  Note that $\hat{E}$ is countably sub-additive, if $C_{V}\left[|\varepsilon |\right]<\infty$, then $\hat{E}\left[|\varepsilon |\right]\leq C_{V}\left[|\varepsilon |\right]<\infty$. Hence, to prove (5), it suffices to prove

(6) $$\begin{array}{c} C_{V}\left[\underset{n}{\sup}{\left(2n\log\log n\right)}^{-1/2}\left|\sum_{k=1}^{n}\varepsilon_{k}\right|\right]<\infty . \end{array}$$

By the definition of $C_{V}$, we have

(7) $$\begin{array}{cc} & \;\;\;\;C_{V}\left[\underset{n}{\sup}{\left(2n\log\log n\right)}^{-1/2}\left|\sum_{k=1}^{n}\varepsilon_{k}\right|\right]\\ & =\int_{0}^{\infty }V\left\{\underset{n}{\sup}\frac{|\sum_{k=1}^{n}\varepsilon_{k}|}{{\left(2n\log\log n\right)}^{1/2}}>x\right\}dx\\ & =\int_{0}^{D}V\left\{\underset{n}{\sup}\frac{|\sum_{k=1}^{n}\varepsilon_{k}|}{{\left(2n\log\log n\right)}^{1/2}}>x\right\}dx+\int_{D}^{\infty }V\left\{\underset{n}{\sup}\frac{|\sum_{k=1}^{n}\varepsilon_{k}|}{{\left(2n\log\log n\right)}^{1/2}}>x\right\}dx\\ & =D+\sum_{l=0}^{\infty }\int_{D}^{\infty }V\left\{\max_{2^{l}\leq n<2^{l+1}}\frac{|\sum_{k=1}^{n}\varepsilon_{k}|}{{\left(2n\log\log n\right)}^{1/2}}>x\right\}dx\\ \; & =D+\sum_{l=0}^{\infty }\int_{D}^{\infty }V\left\{\max_{2^{l}\leq n<2^{l+1}}\left|\sum_{k=1}^{n}\varepsilon_{k}\right|>x{\left(2\cdot 2^{l}\log\log2^{l}\right)}^{1/2}\right\}dx, \end{array}$$

where D>1, value to be determined.

Let $b_{k}={\left(k/\log\log k\right)}^{1/2},\;k\geq 1.$ We define

$$\begin{array}{cc} \; & {\tilde{\varepsilon }}_{k}=\left(-xb_{k}\right)\vee \left(\varepsilon_{k}\wedge xb_{k}\right). \end{array}$$

Noting that

(8) $$\begin{array}{ccc} \sum_{k=1}^{n}\varepsilon_{k} & \leq \sum_{k=1}^{n}{\tilde{\varepsilon }}_{k}+\left|\sum_{k=1}^{n}\left(\varepsilon_{k}-{\tilde{\varepsilon }}_{k}\right)\right|\; & \leq \sum_{k=1}^{n}\left({\tilde{\varepsilon }}_{k}-\hat{E}\left[{\tilde{\varepsilon }}_{k}\right]\right)+\left|\sum_{k=1}^{n}-\hat{E}\left[{\tilde{\varepsilon }}_{k}\right]\right|+\left|\sum_{k=1}^{n}\left(\varepsilon_{k}-{\tilde{\varepsilon }}_{k}\right)\right|. \end{array}$$

According to the Lemma condition $\hat{E}\left[\varepsilon_{1}\right]=\hat{E}\left[-\varepsilon_{1}\right]=0$, we know that

(9) $$\begin{array}{cc} \left|\sum_{k=1}^{n}-\hat{E}\left[{\tilde{\varepsilon }}_{k}\right]\right| & \leq \left|\sum_{k=1}^{n}\left(\hat{E}\left[\varepsilon_{k}\right]-\hat{E}\left[{\tilde{\varepsilon }}_{k}\right]\right)\right|\leq \sum_{k=1}^{n}\hat{E}\left|\varepsilon_{k}-{\tilde{\varepsilon }}_{k}\right|\\ & \leq \sum_{k=1}^{n}\hat{E}\left[\right|\varepsilon_{k}\left|I_{\left\{|\varepsilon_{k}|>xb_{k}\right\}}\right]\\ \; & \leq \sum_{k=1}^{n}\frac{\hat{E}\left[\varepsilon_{k}^{2}\right]}{xb_{k}}\leq c\sum_{k=1}^{n}\frac{1}{xb_{k}}\leq c\cdot \frac{1}{x}{\left(n\log\log n\right)}^{1/2}. \end{array}$$

Then, by (9), and for x>D large enough, we have

(10) $$\begin{array}{c} \max_{2^{l-1}\leq n<2^{l}}\left|\sum_{k=1}^{n}-\hat{E}\left[{\tilde{\varepsilon }}_{k}\right]\right|\leq c\cdot \frac{1}{x}{\left(2^{l+1}\log\log2^{l+1}\right)}^{1/2}\leq \frac{x}{4}{\left(2\cdot 2^{l}\log\log2^{l}\right)}^{1/2}. \end{array}$$

Hence, by (8), we obtain

(11) $$\begin{array}{cc} & \;\;\;\;\sum_{l=0}^{\infty }\int_{D}^{\infty }V\left\{\max_{2^{l}\leq n<2^{l+1}}\sum_{k=1}^{n}\varepsilon_{k}>x{\left(2\cdot 2^{l}\log\log2^{l}\right)}^{1/2}\right\}dx\\ & \leq \sum_{l=0}^{\infty }\int_{D}^{\infty }V\left\{\max_{2^{l}\leq n<2^{l+1}}\sum_{k=1}^{n}\left({\tilde{\varepsilon }}_{k}-\hat{E}\left[{\tilde{\varepsilon }}_{k}\right]\right)>\frac{x}{4}{\left(2\cdot 2^{l+1}\log\log2^{l}\right)}^{1/2}\right\}dx\\ & \;\;\;\;+\sum_{l=0}^{\infty }\int_{D}^{\infty }V\left\{\max_{2^{l}\leq n<2^{l+1}}\left|\sum_{k=1}^{n}\left(\varepsilon_{k}-{\tilde{\varepsilon }}_{k}\right)\right|>\frac{x}{2}{\left(2\cdot 2^{l+1}\log\log2^{l}\right)}^{1/2}\right\}dx\\ \; & :=I_{1}+I_{2}. \end{array}$$

First, to estimate $I_{2}$, by (11), we get

(12) $$\begin{array}{cc} I_{2} & \leq c\sum_{l=0}^{\infty }\int_{D}^{\infty }\frac{\hat{E}\left[\max_{2^{l}\leq n<2^{l+1}}\left|\sum_{k=1}^{n}\left(\varepsilon_{k}-{\tilde{\varepsilon }}_{k}\right)\right|\right]}{x{\left(2^{l}\log\log2^{l}\right)}^{1/2}}dx\\ \; & \leq c\sum_{l=0}^{\infty }\int_{D}^{\infty }\frac{\sum_{k=1}^{2^{l+1}}\hat{E}\left[|\varepsilon_{k}|I_{\left\{|\varepsilon_{k}|>xb_{k}\right\}}\right]}{x{\left(2^{l}\log\log2^{l}\right)}^{1/2}}dx. \end{array}$$

It is important to note that the identical distribution under $\hat{E}$ is defined through continuous functions in $C_{l,Lip}$ and the indicator function of an event is not continuous. We need to modify the indicator function by functions in $C_{l,Lip}$. So, let $g_{\epsilon }$ be a function satisfying that its derivatives of each order are bounded, $g_{\epsilon }\left(x\right)=1$ if x≥1, $g_{\epsilon }\left(x\right)=0$ if x≤1−ϵ, and $0\leq g_{\epsilon }\left(x\right)\leq 1$ for all *x*, where 0<ϵ<1. Then

(13) $$\begin{array}{c} g_{\epsilon }\left(\cdot \right)\in C_{l,Lip}\left(R\right),\;\;I\left\{x\geq 1\right\}\leq g_{\epsilon }\left(x\right)\leq I\left\{x>1-\epsilon \right\}. \end{array}$$

For $\epsilon =\frac{1}{2}$ in (13), by (1) and (12), we have

(14) $$\begin{array}{cc} I_{2} & \leq c\sum_{l=0}^{\infty }\int_{D}^{\infty }\frac{\sum_{k=1}^{2^{l+1}}\hat{E}\left[|\varepsilon_{k}|g\left(\frac{\varepsilon_{k}}{xb_{k}}\right)\right]}{x{\left(2^{l}\log\log2^{l}\right)}^{1/2}}dx\\ & =c\sum_{l=0}^{\infty }\int_{D}^{\infty }\frac{\sum_{k=1}^{2^{l+1}}\hat{E}\left[|\varepsilon_{1}|g\left(\frac{\varepsilon_{1}}{xb_{k}}\right)\right]}{x{\left(2^{l}\log\log2^{l}\right)}^{1/2}}dx\\ \; & \leq c\sum_{l=0}^{\infty }\int_{D}^{\infty }\frac{\sum_{k=1}^{2^{l+1}}\hat{E}\left[|\varepsilon_{1}|I_{\left\{|\varepsilon_{1}|>\frac{1}{2}xb_{k}\right\}}\right]}{x{\left(2^{l}\log\log2^{l}\right)}^{1/2}}dx \end{array}$$

Using condition $\left(A_{1}\right)$. For some δ>0, it is obvious that

(15) $$\begin{array}{cc} \sum_{k=1}^{2^{l+1}}\hat{E}\left[|\varepsilon_{1}|I_{\left\{|\varepsilon_{1}|>\frac{1}{2}xb_{k}\right\}}\right]\; & \leq \sum_{k=1}^{2^{l+1}}\hat{E}\left[\varepsilon_{1}^{2}\left(\log\right|\varepsilon_{1}{\left|\right)}^{1+\delta }\frac{1}{|\varepsilon_{1}\left|(\log\right|\varepsilon_{1}{\left|\right)}^{1+\delta }}\right]I_{\left\{|\varepsilon_{1}|>\frac{1}{2}xb_{k}\right\}}\\ & \leq \hat{E}\left[\varepsilon_{1}^{2}\left(\log\right|\varepsilon_{1}{\left|\right)}^{1+\delta }\right]\sum_{k=1}^{2^{l+1}}\frac{1}{\frac{1}{2}xb_{k}}\cdot \frac{1}{{\left(\log\frac{1}{2}xb_{k}\right)}^{1+\delta }}\\ & \leq c\sum_{k=1}^{2^{l+1}}\sqrt{\frac{\log\log k}{k}}\cdot \frac{1}{{\left(\log k\right)}^{1+\delta }}\cdot \frac{1}{x}\\ \; & \leq c\frac{\sqrt{2^{l+1}\log\log2^{l}}}{{\left(\log2^{l+1}\right)}^{1+\delta }}\cdot \frac{1}{x}. \end{array}$$

Combining (12), (14) and (15), we get

(16) $$\begin{array}{cc} I_{2} & \leq c\sum_{l=0}^{\infty }\int_{D}^{\infty }\frac{1}{x\sqrt{2^{l}\log\log2^{l}}}\cdot \frac{\sqrt{2^{l}\log\log2^{l}}}{{\left(\log2^{l}\right)}^{1+\delta }}\cdot \frac{1}{x}dx\\ & \leq \sum_{l=0}^{\infty }c\int_{D}^{\infty }\frac{1}{x^{2}}dx\cdot \frac{1}{{\left(\log2^{l}\right)}^{1+\delta }}\\ \; & \leq \sum_{l=0}^{\infty }c\frac{1}{{\left(l\log2\right)}^{1+\delta }}<\infty . \end{array}$$

Next, to estimate $I_{1}$. Noting that

(17) $$\begin{array}{cc} I_{1} & \leq \sum_{l=0}^{\infty }\int_{D}^{\infty }V\left\{\max_{l\leq n<2^{l+1}}\sum_{k=1}^{n}\left({\tilde{\varepsilon }}_{k}-\hat{E}\left[{\tilde{\varepsilon }}_{k}\right]\right)>\frac{x}{4}c_{1}{\left(2^{l+1}\log\log2^{l+1}\right)}^{1/2}\right\}dx. \end{array}$$

By the properties of IID random variables, $\left\{{\tilde{\varepsilon }}_{k}-\hat{E}\left[{\tilde{\varepsilon }}_{k}\right]\right\}$ is also a sequence of IID random variables, $\hat{E}\left[{\tilde{\varepsilon }}_{k}-\hat{E}\left[{\tilde{\varepsilon }}_{k}\right]\right]=0,\;\left|{\tilde{\varepsilon }}_{k}-\hat{E}\left[{\tilde{\varepsilon }}_{k}\right]\right|\leq 2xb_{k}\leq 2xb_{2^{l+1}}$ for every *k* and $\hat{E}{\left[{\tilde{\varepsilon }}_{k}-\hat{E}\left[{\tilde{\varepsilon }}_{k}\right]\right]}^{2}\leq 4\hat{E}\left[{\tilde{\varepsilon }}_{k}^{2}\right]=4\hat{E}\left[\varepsilon_{1}^{2}\wedge x^{2}b_{k}^{2}\right]=4{\bar{\sigma }}^{2}<\infty$, $B_{n}∼2^{l+1}\cdot 4{\bar{\sigma }}^{2}$. Taking $y=2xb_{2^{l+1}}$ in (3), then

$$\frac{\frac{x}{4}c_{1}{\left(2^{l+1}\log\log2^{l+1}\right)}^{1/2}}{y}=\frac{c_{1}}{4}\log\log2^{l+1}=A\log\log2^{l+1},\;\;\left(where\;A:=\frac{c_{1}}{4}\right),$$

and

$$\frac{\frac{x}{4}c_{1}{\left(2^{l+1}\log\log2^{l+1}\right)}^{1/2}\cdot y}{B_{n}}=\frac{x^{2}}{4}c_{1}\cdot \frac{1}{4{\bar{\sigma }}^{2}}=\frac{c_{1}x^{2}}{16{\bar{\sigma }}^{2}}=Bx^{2},\;\;\left(where\;B:=\frac{c_{1}}{16{\bar{\sigma }}^{2}}\right).$$

For a sufficiently large *x*, there is a constant $c_{2}<1$, such that $\log\left(1+Bx^{2}\right)\geq c_{2}\log x^{2}$. Choose *D* large enough to make $(Dc_{2}-1)A>1$. And since $D^{\log\log2^{l+1}}=O\left({\left(\log2^{l+1}\right)}^{D}\right)$, using Lemma 2, we have

(18) $$\begin{array}{cc} I_{1} & \leq \sum_{l=0}^{\infty }\int_{D}^{\infty }\exp\left\{A\log\left(\log2^{l+1}\right)\left(1-\log(1+Bx^{2})\right)\right\}dx\\ & \leq \sum_{l=0}^{\infty }{\left(\log2^{l+1}\right)}^{A}\int_{D}^{\infty }\exp\left\{-Ac_{2}\log\log2^{l+1}\log x\right\}dx\\ & \leq c\sum_{l=0}^{\infty }{\left(\log2^{l+1}\right)}^{A}\int_{D}^{\infty }\frac{1}{x^{Ac_{2}\log\log2^{l+1}}}dx\\ & \leq c\sum_{l=0}^{\infty }{\left(\log2^{l+1}\right)}^{A}\frac{1}{{\left(D^{\log\log2^{l+1}}\right)}^{Ac_{2}}}\\ & \leq c\sum_{l=0}^{\infty }{\left(\log2^{l+1}\right)}^{A}\frac{1}{{\left(\log2^{l+1}\right)}^{DAc_{2}}}\\ \; & \leq c\sum_{l=0}^{\infty }\frac{1}{{\left(\log2^{l+1}\right)}^{(Dc_{2}-1)A}}<\infty . \end{array}$$

Combining (11), (16), and (18), we get

$$\begin{array}{c} \sum_{l=0}^{\infty }\int_{D}^{\infty }V\left\{\max_{2^{l}\leq n<2^{l+1}}\sum_{k=1}^{n}\varepsilon_{k}>x{\left(2\cdot 2^{l}\log\log2^{l}\right)}^{1/2}\right\}dx<\infty . \end{array}$$

For $\left(-\sum_{k=1}^{n}\varepsilon_{k}\right)$, we have the same convergence as the above. Then, we obtain

(19) $$\begin{array}{c} \sum_{l=0}^{\infty }\int_{D}^{\infty }V\left\{\max_{2^{l}\leq n<2^{l+1}}\left|\sum_{k=1}^{n}\varepsilon_{k}\right|>x{\left(2\cdot 2^{l}\log\log2^{l}\right)}^{1/2}\right\}dx<\infty . \end{array}$$

From (7) and (19), (6) holds. So Lemma is proved.  □

**Proof of Theorem 1.**  For m,n,t∈N, define

$$\begin{array}{cc} \; & Y_{m,n}=\frac{1}{a_{n}}\sum_{t=1}^{n}\sum_{j=-m}^{m}\alpha_{j}\varepsilon_{t-j},\\ \; & {\tilde{\alpha }}_{m}=0,\;\;\;{\tilde{\alpha }}_{j}=\sum_{i=j+1}^{m}\alpha_{i},\;\;\;j=0,1,\cdot \cdot \cdot ,m-1,\\ \; & {\tilde{\tilde{\alpha }}}_{-m}=0,\;\;\;{\tilde{\tilde{\alpha }}}_{j}=\sum_{i=-m}^{j-1}\alpha_{i},\;\;\;j=-m+1,-m+2,\cdot \cdot \cdot ,0,\\ \; & \tilde{\varepsilon_{t}}=\sum_{j=0}^{m}{\tilde{\alpha }}_{j}\varepsilon_{t-j},\;\;\;{\tilde{\tilde{\varepsilon }}}_{t}=\sum_{j=-m}^{0}{\tilde{\tilde{\alpha }}}_{j}\varepsilon_{t-j}. \end{array}$$

Obviously, we have

(20) $$\begin{array}{cc} Y_{m,n} & =\left(\sum_{j=-m}^{m}\alpha_{j}\right)\frac{1}{a_{n}}\left(\sum_{t=1}^{n}\varepsilon_{t}\right)+\frac{1}{a_{n}}\left({\tilde{\varepsilon }}_{0}-{\tilde{\varepsilon }}_{n}+{\tilde{\tilde{\varepsilon }}}_{n+1}-{\tilde{\tilde{\varepsilon }}}_{1}\right), \end{array}$$

(21) $$\begin{array}{c} \frac{1}{a_{n}}\sum_{t=1}^{n}X_{t}=Y_{m,n}+\frac{1}{a_{n}}\left(\sum_{t=1}^{n}\sum_{|j|>m}\alpha_{j}\varepsilon_{t-j}\right). \end{array}$$

First note that

$$\frac{{\tilde{\varepsilon }}_{0}}{a_{n}}={\left(2n\log\log n\right)}^{-1/2}\sum_{j=0}^{m}\sum_{i=j+1}^{m}\alpha_{i}\varepsilon_{-j}\to 0\;\;a.s.\;V,\;\;n\to \infty ,$$

and

$$\frac{{\tilde{\tilde{\varepsilon }}}_{1}}{a_{n}}={\left(2n\log\log n\right)}^{-1/2}\sum_{j=-m}^{0}\sum_{i=-m}^{j-1}\alpha_{i}\varepsilon_{1-j}\to 0\;\;a.s.\;V,\;\;n\to \infty .$$

For any δ>0, the Lemma 4.5 in Zhang [13] shows that

$$\begin{array}{c} \sum_{n=1}^{\infty }V(|\varepsilon_{1}\left|>\delta a_{n}\right)<\infty ⟺C_{V}\left[\frac{|\varepsilon_{1}{|}^{2}}{\log\log|\varepsilon_{1}|}\right]<\infty . \end{array}$$

Hence by (1), $\left(A_{3}\right)$ and let $g_{\epsilon }\left(\cdot \right)$ be a smooth function satisfying (13), for any δ>0

$$\begin{array}{cc} \sum_{n=1}^{\infty }V(|\varepsilon_{n-j}\left|/a_{n}>\delta \right)\; & \leq \sum_{n=1}^{\infty }\hat{E}\left[g_{\frac{1}{2}}\left(\frac{|\varepsilon_{n-j}|}{a_{n}\delta }\right)\right]\\ \; & =\sum_{n=1}^{\infty }\hat{E}\left[g_{\frac{1}{2}}\left(\frac{|\varepsilon_{1}|}{a_{n}\delta }\right)\right]\;\;\left(since\;\varepsilon_{n-j}\overset{d}{=}\varepsilon_{1}\right)\\ \; & \leq \sum_{n=1}^{\infty }V(|\varepsilon_{1}\left|>\frac{1}{2}\delta a_{n}\right)\\ \; & \leq cC_{V}\left[\frac{|\varepsilon_{1}{|}^{2}}{\log\log|\varepsilon_{1}|}\right]<\infty . \end{array}$$

By the Lemma 1 (Borel–Cantelli Lemma), we have

$$V(\underset{n}{lim sup}\frac{|\varepsilon_{n-j}|}{a_{n}}>\delta )=0,\;\;\;\;\forall \delta >0.$$

So we get

$$V(\underset{n}{lim sup}\frac{|\varepsilon_{n-j}|}{a_{n}}\leq \delta )=1,\;\;\;\;\forall \delta >0.$$

Thus

$$\frac{{\tilde{\varepsilon }}_{n}}{a_{n}}={\left(2n\log\log n\right)}^{-1/2}\sum_{j=0}^{m}\sum_{i=j+1}^{m}\alpha_{i}\varepsilon_{n-j}\to 0\;\;a.s.\;V,\;\;n\to \infty .$$

Using the proof similar to the above formula, we get

$$\frac{{\tilde{\tilde{\varepsilon }}}_{n+1}}{a_{n}}={\left(2n\log\log n\right)}^{-1/2}\sum_{j=-m}^{0}\sum_{i=-m}^{j-1}\alpha_{i}\varepsilon_{n+1-j}\to 0\;\;a.s.\;V,\;\;n\to \infty .$$

So, we conclude that

(22) $$\begin{array}{c} \frac{1}{a_{n}}\left({\tilde{\varepsilon }}_{0}-{\tilde{\varepsilon }}_{n}+{\tilde{\tilde{\varepsilon }}}_{n+1}-{\tilde{\tilde{\varepsilon }}}_{1}\right)\to 0\;\;a.s.\;V,\;\;n\to \infty . \end{array}$$

Combining with (20), (21), and (22), we have

(23) $$\begin{array}{cc} \underset{n}{lim sup}\frac{|T_{n}|}{a_{n}}\; & =\underset{n}{lim sup}|Y_{m,n}+\sum_{|j|>m}\alpha_{j}\frac{1}{a_{n}}\sum_{t=1}^{n}\varepsilon_{t-j}|\\ & \leq \underset{n}{lim sup}|\sum_{j=-m}^{m}\alpha_{j}|\frac{1}{a_{n}}|\sum_{t=1}^{n}\varepsilon_{t}|+\underset{n}{lim sup}\sum_{|j|>m}\left|\alpha_{j}\right|\frac{1}{a_{n}}|\sum_{t=1}^{n}\varepsilon_{t-j}|\\ \; & \leq \underset{n}{lim sup}|\sum_{j=-m}^{m}\alpha_{j}|\frac{1}{a_{n}}|\sum_{t=1}^{n}\varepsilon_{t}|+\sum_{|j|>m}\left|\alpha_{j}\right|\underset{n}{\sup}\frac{1}{a_{n}}|\sum_{t=1}^{n}\varepsilon_{t-j}|. \end{array}$$

By the stationariness of $\left\{\varepsilon_{k}\right\}$ and the Lemma 4, we have

$$\begin{array}{c} \hat{E}\left[\underset{n}{\sup}{\left(2n\log\log n\right)}^{-1/2}\left|\sum_{t=1}^{n}\varepsilon_{t-j}\right|\right]=\hat{E}\left[\underset{n}{\sup}{\left(2n\log\log n\right)}^{-1/2}\left|\sum_{t=1}^{n}\varepsilon_{t}\right|\right]<\infty , \end{array}$$

then

(24) $$\begin{array}{c} \underset{n}{\sup}{\left(2n\log\log n\right)}^{-1/2}\left|\sum_{t=1}^{n}\varepsilon_{t-j}\right|<\infty . \end{array}$$

The countably sub-additive of $\hat{E}$ shows that V is countably sub-additive. Then, according to the condition of Lemma 3, $\left\{\varepsilon_{i}\right\}$ satisfies (4). Next, using (4) and (24), let m→∞ in (23), we get

(25) $$\begin{array}{c} \underset{n\to \infty }{lim sup}\frac{|T_{n}|}{a_{n}}\leq |\sum_{j=-\infty }^{\infty }\alpha_{j}|\bar{\sigma }\;\;\;\;a.s.\;\;V. \end{array}$$

So, we obtain

$$\begin{array}{c} V\left(\underset{n\to \infty }{lim sup}\frac{|T_{n}|}{a_{n}}\leq A\bar{\sigma }\right)=1. \end{array}$$

The proof of Theorem 1 now completes.  □

## 4. Conclusions

This paper mainly studies the LIL of linear processes under capacity induced by sub-linear expectation, which is based on Zhang [13]. According to the new concepts of distribution and independence under Peng’s framework, we define the strictly stationary sequence under sub-linear expectation, and further redefine the linear processes under sub-linear expectation. We first obtain Lemma 4 by truncating random variables, countably sub-additive of capacity and exponential inequality under sub-linear expectation. Secondly, the tail of the partial sum of linear processes tends to zero in the sense of capacity by using the decomposition of the partial sum of linear processes, Lemma 4, the transformation of Choquet expectation and integral. Finally, the main results of this paper are obtained by using Lemma 3.

The results obtained in this paper enrich the limit theory of capacity (non additive probability) and are also a natural generalization of the LIL under classical additive probability. The key to the main results of this paper is an exponential inequality. If we can establish the corresponding exponential inequalities for negative dependent (ND) sequences, then we can obtain the LIL of linear processes generated by stationary ND sequences under sub-linear expectation. ND sequences are weaker than independent sequences. Therefore, it is an impending problem to study the theoretical properties of ND sequences in sub-linear expectation, which is the subject of future research. 

## Data Availability

Not applicable.
